# High evolutionary potential of marine zooplankton

**DOI:** 10.1002/ece3.644

**Published:** 2013-06-26

**Authors:** Katja T C A Peijnenburg, Erica Goetze

**Affiliations:** 1 Institute for Biodiversity and Ecosystem Dynamics, University of AmsterdamP.O. Box 94248, 1090 GE, Amsterdam, The Netherlands; 2 Department Marine Zoology, Naturalis Biodiversity CenterP.O. Box 9517, 2300 RA, Leiden, The Netherlands; 3 Department of OceanographySchool of Ocean and Earth Science and Technology, University of Hawaii at ManoaHonolulu, Hawaii, 96822

**Keywords:** Adaptation, climate change, marine, oceanic, selection, zooplankton

## Abstract

Open ocean zooplankton often have been viewed as slowly evolving species that have limited capacity to respond adaptively to changing ocean conditions. Hence, attention has focused on the ecological responses of zooplankton to current global change, including range shifts and changing phenology. Here, we argue that zooplankton also are well poised for *evolutionary responses* to global change. We present theoretical arguments that suggest plankton species may respond rapidly to selection on mildly beneficial mutations due to exceptionally large population size, and consider the circumstantial evidence that supports our inference that selection may be particularly important for these species. We also review all primary population genetic studies of open ocean zooplankton and show that genetic isolation can be achieved at the scale of gyre systems in open ocean habitats (100s to 1000s of km). Furthermore, population genetic structure often varies across planktonic taxa, and appears to be linked to the particular ecological requirements of the organism. In combination, these characteristics should facilitate adaptive evolution to distinct oceanographic habitats in the plankton. We conclude that marine zooplankton may be capable of rapid evolutionary as well as ecological responses to changing ocean conditions, and discuss the implications of this view. We further suggest two priority areas for future research to test our hypothesis of high evolutionary potential in open ocean zooplankton, which will require (1) assessing how pervasive selection is in driving population divergence and (2) rigorously quantifying the spatial and temporal scales of population differentiation in the open ocean.

Recent attention has focused on the ecological responses of open ocean zooplankton to current global change, including range shifts and changing phenology. Here, we argue that marine zooplankton also are well poised for evolutionary responses to global change.

## Evolution in the Open Sea

**Figure 1:**
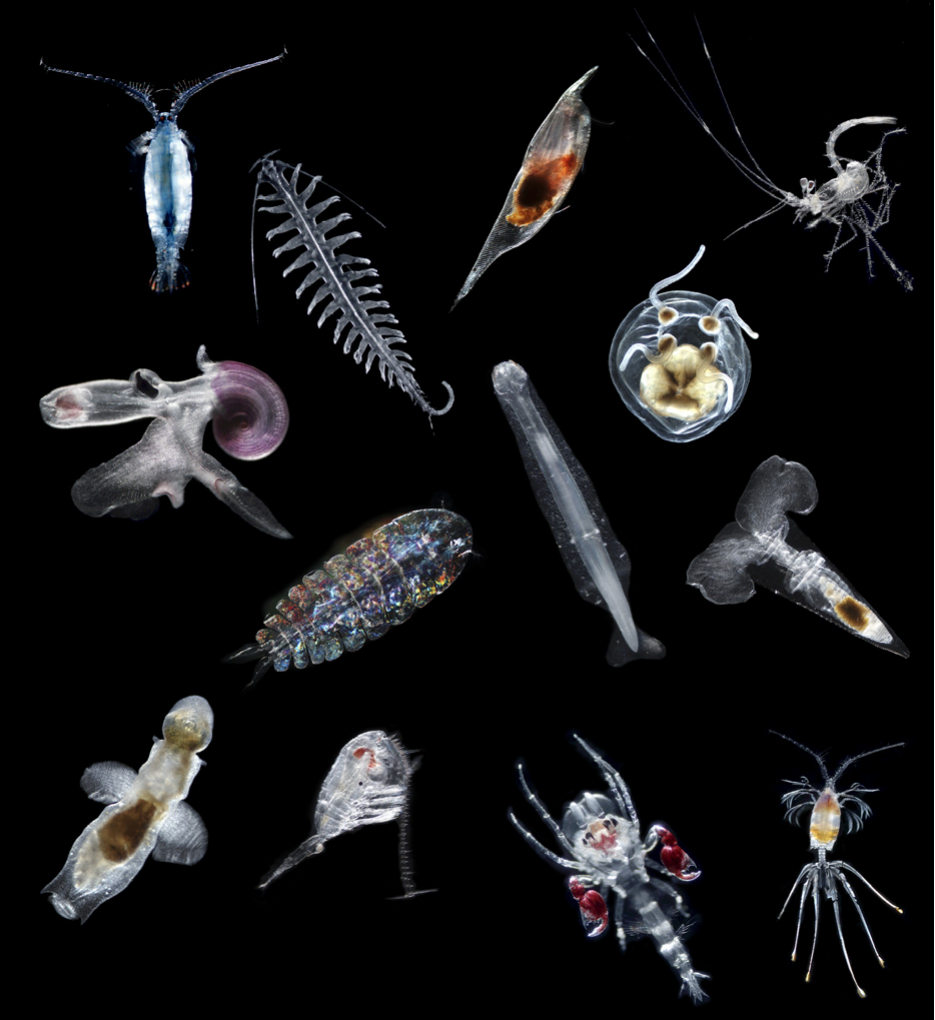
Examples of the diverse holozooplankton assemblage of the Atlantic ocean (members of the phyla Arthropoda, Mollusca, Annelida, Cnidaria, and Chaetognatha are represented). Photographed by the authors during the Atlantic Meridional Transect (AMT22) research cruise in October–November 2012.

The oceans are changing on a global scale and, in some cases, at rates greatly exceeding those observed in the historical and recent geological record (e.g., Pelejero et al. 124). Holoplankton, the organisms that spend their entire life cycle in the open water column, are particularly good indicators of climate change (Hays et al. 74), and show the most dramatic range shifts of any organisms reported in either terrestrial or marine environments (e.g., Beaugrand et al. 12, 14; Burrows et al. 32). Marine zooplankton serve as key links in the food web between primary producers and higher trophic levels (e.g., fish, micronekton), and also are important mediators of biogeochemical fluxes in the ocean. Marine zooplankton are a phylogenetically diverse group (see Fig. 1) including representatives from 12 animal phyla (Angel 3). Most taxa are diploid and sexual species, but some notable exceptions include members of the phyla Cnidaria and Urochordata, which alternate between asexual and sexual phases of their life cycle (e.g., scyphozoans, salps).

Although we know very little about the evolutionary potential of open ocean zooplankton, most authors have explicitly or implicitly assumed that zooplankton will show limited evolutionary responses to climate change (e.g., Helaouët and Beaugrand 75; Reygondeau and Beaugrand 134; Stegert et al. 146; but see Dam 43). Hence, much attention has been focused on the observations of ecological responses to climate change in these species, for example, on their changing species' distributions and phenology (e.g., Hays et al. 74; Richardson 136; Ji et al. 82). Here, we consider evolutionary responses, that is, those that result in genetic changes in a population, for example, in response to selective pressure. Although the number of studies that have rigorously tested for genetic adaptation in marine zooplankton is small, and limited to estuarine and coastal taxa that are amenable to laboratory experimentation (reviewed in Dam 43), unequivocal evidence exists for genetic adaptation in marine zooplankton. One example is that grazer populations with a history of exposure to toxic algal blooms have significantly higher fitness when challenged with toxic prey than those with little or no such history of exposure (Colin and Dam 37). There is also an extensive literature providing evidence for adaptive evolution in freshwater zooplankton, for example, using the water flea *Daphnia* as a model system (e.g., Critescu et al. 41; Orsini et al. 114). Our review focuses on the open ocean zooplankton, the majority of which cannot be cultured in the laboratory. We argue that evolutionary responses to global change are important to consider for these taxa, and explore indirect methods for studying evolution in these oceanic species.

Three primary arguments have traditionally supported the perception that open ocean zooplankton have limited capacity to evolve in comparison to other terrestrial and marine species (e.g., van der Spoel and Pierrot-Bults 145; Angel 2; Palumbi 116; Grosberg and Cunningham 70). First, low species diversity in the plankton has been interpreted as evidence for depressed speciation rates in comparison to other organisms. The pelagic habitat lacks obvious geographic isolating barriers that would be necessary for speciation in allopatry, the most common geographic mode of speciation (Pierrot-Bults and van der Spoel 127; Angel 3; van der Spoel 144; Norris 110; Coyne and Orr 40). Although molecular studies are revealing cryptic species across the spectrum of pelagic animal phyla (Dawson and Jacobs 50; Darling and Wade 44; Morard et al. 104; Goetze 65; Jennings et al. 81; Miyamoto et al. 103; Ortman et al. 115), true estimates of species numbers are unlikely to be orders of magnitude higher than current estimates. A recent estimate of global marine diversity reported that ~226,000 eukaryotic marine species are described, of a total of 0.7–1.0 million marine species (Appeltans et al. 4). Of these, only a small fraction are DNA barcoded (Bucklin et al. 31), and molecular methods are estimated to add tens of thousands, rather than hundreds of thousands, of species to the currently accepted species list (Appeltans et al. 4). Thus, we can accept that species diversity in the plankton is relatively low. Second, marine zooplankton have among the largest (effective) population sizes of any organisms on Earth (e.g., Lynch et al. 93; Bucklin and Wiebe 23; Avise 6; Goetze 64; Peijnenburg et al. 122), and therefore genetic drift is expected to be ineffective at changing allele frequencies within these populations. Third and finally, marine plankton are envisioned to be high-dispersal species (Ekman 52; Angel 3; van der Spoel 144), with extensive migration among populations limiting their capacity for local adaptation. Plankton have been likened to airborne spores or wind-dispersed seeds that can drift almost anywhere in the ocean (Norris 110), with their biogeographic ranges limited only by their ability to find suitable habitat for the establishment of new populations (Norris and de Vargas 111; Sexton and Norris 142). Collectively, these three arguments have supported a persistent view of low evolutionary potential for marine zooplankton, in comparison to other marine and terrestrial groups. However, a number of recent observations and insights suggest that we may have overlooked important processes driving the evolution of open ocean zooplankton. Here, we propose the idea that selection may be a dominant driver of marine zooplankton evolution based on theoretical insights and circumstantial evidence that selection may be widespread in these species. We also review the 46 primary population genetic and phylogeographic studies that focus on population-level differentiation in marine zooplankton (55 taxa; 001), and show that genetic isolation often is observed across distinct pelagic biomes even in these entirely planktonic taxa.

**Table 1:** Summary of all 46 population genetic and phylogeographic studies that focus on open ocean zooplankton

Species name	Geographic area	Marker type	Sample size	Presence and scale of structure	Pairwise *F*_ST_ (range)	h	pi	Dev. neutr.?	Reference
Planktonic copepods									
*Labidocera aestiva*	W. North Atlantic, US coast	Allozymes (6 loci)	211	Regional, within NW Atlantic	n.r.	N/A	N/A	N/A	Bucklin and Marcus (21)
*Calanus australis*	Tropical and subtropical Pacific	Allozymes (2 loci)	328	Panmixia, but high variation in this species	n.r.	N/A	N/A	N/A	Afanas'yev et al. (1)
*Undinula darwinii*	Tropical and subtropical Pacific	Allozymes (2 loci)	742	Isolation by distance observed (over 3000 km)	n.r.	N/A	N/A	N/A	Afanas'yev et al. (1)
*Metridia pacifica*	California Current	Allozymes (7 polymorphic loci)	n.r., >420	Genetic heterogenity, weak or absent structure	0.011–0.141	N/A	N/A	N/A	Bucklin et al. (24)
*Metridia pacifica*	California Current	Allozymes (6 loci)	3040	Genetic heterogenity, some structure at mesoscale	n.r.	N/A	N/A	N/A	Bucklin (19)
*Calanus finmarchicus*	W. North Atlantic	mtDNA sequence (16S rRNA)	182^1^	Genetic heterogenity, but not structured	n.r.	n.r.	0.0042	n.r.	Bucklin and Kocher (20)
*Calanus finmarchicus*	Gulf of Maine	Allozymes, mtDNA RFLPs	628	Panmixia	0.021–0.039	N/A	n.r.	N/A	Kann and Wishner (83)
*Calanus finmarchicus*	W. North Atlantic and Norwegian Sea	mtDNA sequence (16S rRNA)	104^1^	Weak structure between NW Atlantic and Norwegian Sea	n.r.	0.670	0.0061	n.r.	Bucklin et al. (25)
*Nannocalanus minor*	NW and NE Atlantic	mtDNA sequence (16S rRNA)	155^1^	Panmixia within Types I and II	n.r.	0.880	0.0232	n.r.	Bucklin et al. (26)
*Nannocalanus minor*	W. subtropical North Atlantic	mtDNA sequence (16S rRNA)	158	n.r.	n.r.	0.824	0.0050	n.r.	Bucklin and Wiebe (23)
*Calanus finmarchicus*	Boreal North Atlantic	mtDNA sequence (16S rRNA)	216	n.r.	n.r.	0.368	0.0037	n.r.	Bucklin and Wiebe (23)
*Calanus finmarchicus*	Boreal North Atlantic	Nuclear SNPs (2 loci)+ and DNA sequence	92^1^	Regional, among Iceland samples	n.r.	n.r.	n.r.	n.r.	Bucklin et al. (28)
*Acartia clausi*	Five Norwegian fjords (NE Atlantic)	mtDNA sequence (16S rRNA)	96	Between Norwegian Fjords	n.r.	0.674	0.0026	n.r.	Bucklin et al. (29)
*Calanus helgolandicus, C. euxinus*	NE Atlantic, Mediterranean, Black Sea	mtDNA sequence (COI, cyt b)	72^1^	Between basins	0.000–0.524	0.703	0.0028	Yes	Papadopoulos et al. (118)
*Eucalanus spinifer*	Global	mtDNA sequence (COI)	383^1^	Between basins and central gyres	0.000–0.587	0.487	0.0022	Yes	Goetze (64)
*Eucalanus hyalinus*	Global	mtDNA sequence (COI)	450	Between basins and central gyres	0.000–0.826	0.887	0.0276	Yes	Goetze (64)
*Calanus helgolandicus, C. euxinus*	NE Atlantic, Mediterranean, Black Sea	mtDNA sequence (COI)	99^1^	Regional, between basins, European Seas	0.316–0.509	0.860	n.r.	n.r.	Unal et al. (155)
*Macrosetella gracilis*	North Pacific, North Atlantic	mtDNA sequence (COI)	149^1^	Within and between basins	0.117–0.235	0.899	0.0168	No	Eberl et al. (51)
*Calanus pacificus*	Boreal North Pacific	mtDNA sequence (COI)	398	Between coastal and open ocean sites	0.060–0.750	0.912	0.0089	n.r.	Nuwer et al. (113)
*Calanus finmarchicus*	Boreal North Atlantic	mtDNA sequence, Microsats	313	Panmixia	n.r.	n.r.	n.r.	n.r.	Provan et al. (131)
*Calanus glacialis*	N Atlantic, Arctic, N Pacific	mtDNA sequence (16S rRNA)	443^1^	Strong structure between Pacific and Arctic Ocean	0.000–0.680	0.295	n.r.	n.r.	Nelson et al. (105)
*Disseta palumbii*	equatorial W Pacific, marginal seas	AFLPs	34^1^	Between Sulu Sea vs other regions (clade B)	0.000–0.018	0.236	0.0208	n.r.	Machida and Nishida (95)
*Calanus finmarchicus*	Boreal North Atlantic	Nuclear SNPs (3 loci)	351^1^	Weak structure, within and between regions	0.000–0.240	n.r.	n.r.	n.r.	Unal and Bucklin (154)
*Subeucalanus pileatus*	Global	mtDNA sequence (16S rRNA)	204	Within and between basins	0.000–0.997	0.439	0.0023	n.r.	Goetze (66)
*Clausocalanus lividus*	North Pacific, North Atlantic	mtDNA sequence (COI)	87^1^	Clade divergence, between basins	0.000–1.000	0.874	0.0337	No	Blanco-Bercial et al. (16)
*Clausocalanus arcuicornis*	Global	mtDNA sequence (COI)	96^1^	Within and between basins	0.0618–0.301	0.958	0.0180	Yes	Blanco-Bercial et al. (16)
*Acartia tonsa – lineage X*	W. North Atlantic, US coast	mtDNA (COI) and nucDNA (ITS1) sequence	88	Little geographic structure, invasive	n.r.	0.620 (mt)	0.0024	No	Chen and Hare (35)
*Acartia tonsa – lineage F*	W. North Atlantic, US coast	mtDNA (COI) and nucDNA (ITS1) sequence	104	Regional	n.r.	0.974 (mt)	0.0290	No	Chen and Hare (35)
*Acartia tonsa - lineage S*	W. North Atlantic, US coast	mtDNA (COI) and nucDNA (ITS1) sequence	132	Regional	n.r.	0.738 (mt)	0.0055	Yes	Chen and Hare (35)
*Calanus helgolandicus, C. euxinus*	North Atlantic, European Seas	mtDNA sequence (16S rRNA)	316^1^	Within and between basins, European Seas	0.000–0.744	0.529	0.0033	Yes	Yebra et al. (163)
*Pleuromamma xiphias*	Global	mtDNA sequence (COI)	651	Within and between basins, >100s km	0.000–0.793	0.799	0.0136	Yes	Goetze (67)
*Haloptilus longicornis*	Global	mtDNA sequence (COII)	1059	Within and between basins, >100s km	0–0.46	0.800	0.0200	Yes	Norton and Goetze (in press)
Other crustaceans									
*Euphausia superba*	Weddell Sea, Scotia Sea, Antartic Peninsula	Allozymes (7 polymorphic loci)	381	Panmixia	n.r.	N/A	N/A	N/A	Schneppenheim and Macdonald (139)
*Euphausia krohnii*	W. North Atlantic, US coast, slope	Allozymes (8 polymorphic loci)	95^1^	Genetic heterogeneity, but not structured	n.r.	N/A	N/A	N/A	Bucklin and Wiebe (22)
*Nematocelis megalops*	W. North Atlantic, US coast, slope	Allozymes (7 polymorphic loci)	161	Genetic heterogeneity, but not structured	n.r.	N/A	N/A	N/A	Bucklin and Wiebe (22)
*Euphausia crystallorophias*	Bransfield St, Elephant Is, Wedell Sea	Allozymes (6 polymorphic loci)	612	Panmixia	n.r.	N/A	N/A	N/A	Kuhl and Schneppenheim (87)
*Euphausia superba*	Bransfield St, Elephant Is, Wedell Sea	Allozymes (8 polymorphic loci)	1044	Panmixia	n.r.	N/A	N/A	N/A	Kuhl and Schneppenheim (87)
*Euphausia superba*	Circumpolar, Southern Ocean	Allozymes (8 polymorphic loci)	880	Panmixia	0.000–0.004	N/A	N/A	N/A	Fevolden and Scheppenheim (56)
*Meganyctiphanes norvegica*	Norwegian and Greenland Seas	Allozymes (5 polymorphic loci)	1043	Panmixia	n.r.	N/A	N/A	N/A	Sundt and Fevolden (148)
*Meganyctiphanes norvegica*	North Atlantic	mtDNA sequence (COI, cyt b)	101^1^	Between Norwegian Sea and NW Atlantic, basin scale	n.r.	0.685 (COI), 0.908 (cyt b)	0.0038 (COI), 0.0182 (cyt b)	n.r.	Bucklin et al. (27)
*Euphausia superba*	Ross Sea to Wedell Sea (4 sites)	mtDNA sequence (ND1)	249	South Georgia distinct from Wedell Sea	0.000–0.021	0.850	0.0138	Yes	Zane et al. (164)
*Meganyctiphanes norvegica*	NE Alantic and Mediterranean Sea	mtDNA sequence (ND1), SSCP	1385	Between basins, European Seas	0.000–0.641	0.560	0.0038	No	Zane et al. (165)
*Euphausia crystallorophias*	Davis Sea to WA Peninsula (3 regions)	mtDNA sequence (COI), SSCP	232	Genetic heterogeneity, but not structured	0.027–0.087	n.r.	n.r.	Yes	Jarman et al. (80)
*Nematoscelis difficilis*	California Current	mtDNA sequence (COI)	149	Panmixia	n.r.	0.794	n.r.	n.r.	Bucklin et al. (30)
*Meganyctiphanes norvegica*	Boreal and subarctic N. Atlantic, European Seas	mtDNA sequence (ND1), SSCP	982	Primarily between basins, European Seas	0.000–0.128	0.445	0.0050	n.r.	Papetti et al. (119)
*Euphausia superba*	Scotia Sea, distinct swarms	mtDNA sequence (COI)	504	Panmixia	0.000–0.022	0.999	0.0110	Yes	Goodall-Copestake et al. (68)
*Euphausia superba*	Western Antarctic Peninsula	mtDNA SNPs (in cyt b, 4 sites)	585^1^	Weak or absent spatial structure, temporal differentiation	n.r.	n.r.	n.r.	n.r.	Batta-Lona et al. (8)
*Euphausia superba*	Circumpolar, Southern Ocean	mtDNA sequence (ND1), Microsats	660	Panmixia	0.000–0.024	0.856	0.0139	Yes	Bortolotto et al. (17)
Chaetognaths									
*Parasagitta elegans*	Japanese coastal waters	Allozymes (8 polymorphic loci)	194	Weak structure between Sea of Japan and Oyashio	n.r.	N/A	N/A	N/A	Thuesen et al. (152)
*Sagitta setosa*	NE Atlantic, Mediterranean, Black Sea	mtDNA sequence (COII)	82^1^	Strong structure, between basins	n.r.	1.000	0.0221	Yes	Peijnenburg et al. (121)
*Sagitta elegans*	North East Atlantic	mtDNA sequence (COII)	37^1^	Panmixia	0.000–0.177	1.000	0.0612	Yes	Peijnenburg et al. (122)
*Sagitta setosa*	North East Atlantic	mtDNA sequence (COII)	32^1^	Panmixia	0.000–0.126	1.000	0.0208	Yes	Peijnenburg et al. (122)
*Sagitta setosa*	NE Atlantic, Mediterranean, Black Sea	mtDNA RFLP (COII), Microsats	1739	Strong structure, between basins	0.000–0.827 (mt), 0.000–0.037 (nuc)	0.370	0.009	n.r.	Peijnenburg et al. (123)
Cnidaria									
*Pelagia noctiluca*	E Atlantic, Mediterranean Sea	mtDNA (COI) and nucDNA (ITS1, ITS2) sequence	144	No structure, probable admixture between Med and Atl	0.000 - 0.095 (mt), 0.000 - 0.004 (nuc)	0.96 (mt), 0.723 (nuc)	0.0116 (mt), 0.0031 (nuc)	Yes	Stopar et al. (147)
Ctenophora									
*Mnemiopsis leidyi*	NW Atlantic, Gulf of Mexico (non native areas: Eurasia)	Microsatellites (6 loci used)	467	Between two source populations (New England, Gulf of Mexico)	0.000–0.268	N/A	N/A	n.r.	Reusch et al. (133)

Only studies that address population subdivision and genetic structure are included. Columns are: Species name, including specific genetic lineages if relevant; Geographic area, the geographic coverage of sampling; Marker type, the genetic marker(s) used to infer population structure; Sample size, for the species listed only, in allozyme studies this is the maximum number of alleles for any locus/2 (typically reported as No. alleles surveyed, not individuals); Presence and Scale of Structure, the geographic scale over which population structure was inferred to occur; Pairwise *F*_ST_, range of *F*_ST_ values among individual population samples; h, haplotype diversity; pi, nucleotide diversity; Neutr?, if significant deviations from neutrality were observed in Tajima's *D*, Fu and Li's, or Rozas's *R*^2^ tests. Note that calculations of h, pi, and neutrality tests are only applicable to mtDNA markers because these are haploid and hence gametic phase is known. N/A, not applicable; n.r., not reported; NS, nonsignificant.

In the sample size column indicates studies in which over 1/4 of the population samples had *N* < 15 individuals sampled. Inferences of population structure may be influenced by low sample size in these studies.

## Selection as a Dominant Evolutionary Force

**Figure 2:**
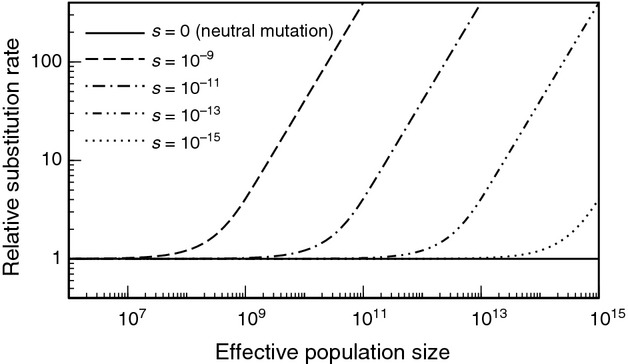
Selection in large plankton populations. Results from a model derived from standard population genetics theory (Crow and Kimura 42) showing that substitution rate is sensitive to small selection coefficients in large populations. For slightly beneficial mutations with selection coefficient (*s*), the fixation probability (*P*) can be approximated by: 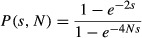 where *N* is diploid effective population size. When *s* converges to 0 (i.e., mutations are neutral) *P* is 1/2*N* and as *s* grows larger *P* becomes approximately 2*s*. For simplicity we assume that the substitution rate can be described as the number of mutations arising times the fixation probability. The substitution rate, relative to the neutral substitution rate, is plotted as a function of effective population size for various immeasurably small selection coefficients (ranging from 10^−9^ to 10^−15^). This model ignores clonal interference, that is, competition between beneficial mutations, which is expected to slow down the response to selection in asexual species (e.g., Gerrish and Lenski 59).

One important misinterpretation that has supported the view of low evolutionary potential in marine zooplankton is the relative unimportance of genetic drift in influencing allele frequencies in large populations, according to standard population genetic models (Crow and Kimura 42). However, relatively little attention has been paid to the reverse side of this coin, namely that selection is highly effective in large populations. The reasons for the higher efficacy of selection are twofold. First, more adaptive mutations occur in large populations, simply because there are more individuals to undergo mutation (e.g., Lynch et al. 93; Barton 7). Furthermore, beneficial mutations are common enough in large populations to arise both recurrently and on independent genetic backgrounds, increasing the chance that they arrive at the right time, place, and on the appropriate genetic background (Pennings and Hermisson 125,b; Ralph and Coop 132). Second, selection is more effective because the stochastic effect of drift is smaller in large populations (e.g., Gillespie 61, 62, 63; Charlesworth 34). A simple theoretical model (Fig. 2) demonstrates how even very mild, immeasurably small, selection pressures (*s* = 10^−9^ to 10^−15^) can quickly drive beneficial mutations to fixation at population sizes typical of marine zooplankton (*N*_e_ = 10^7^ to 10^15^; Fig. 2). Figure 2 also shows that with increasingly large populations, the selection coefficients that have a substantial effect with respect to substitution rate become progressively smaller. Therefore, the assumption of neutrality will be more likely violated in species with large populations. Even though there is still active debate as to whether most of the genetic variation within and between species is selectively neutral (Kimura 85, 86), or whether a large proportion of the variation is subject to selection (Gillespie 60; Hahn 71; Wares 161), we argue here that selection may be a dominant force in the evolution of open ocean plankton, because they are at the extreme end of the scale in terms of population size of marine organisms. The effect of population size is illustrated by genome-wide studies in terrestrial model organisms that reported substantial evidence of adaptive evolution in *Drosophila* and *Escherichia coli* (large populations), whereas genetic variation in *Homo sapiens* and *Arabidopsis* (small populations) conformed to a background selection model, with a large number of segregating deleterious polymorphisms (reviewed in Hahn 71). Barton (7) and Karasov et al. (84) have also argued that adaptation is not mutation limited in very large populations, and that rapid appearance of adaptive alleles can enable fast evolution, for example, adaptation to insecticide in *Drosophila* within 50 years, or ~1000 generations.

Thus far, no studies of open ocean zooplankton have directly addressed the question as to how pervasive selection is in driving evolutionary change in natural populations (but see Dam 43 and Sanford and Kelly 138 for selected examples from coastal zooplankton and benthic invertebrates with meroplanktonic larvae). One interesting conclusion from Sanford and Kelly (138) was that species with planktonic dispersal comprise a surprisingly high percentage (66%) of the marine invertebrates known or suspected of exhibiting local adaptation. Several studies of pelagic marine fish have indicated a much more important role for selection than was previously thought (e.g., Hauser and Carvalho 73; Gaggiotti et al. 57; Bradbury et al. 18; but see McCusker and Bentzen 98), and selection experiments with the coccolithophore *Emiliania huxleyi* have shown that adaptive evolution to ocean acidification can occur within ~500 generations (Lohbeck et al. 91).

Three lines of evidence suggest that selection may be a particularly important driver of evolution in open ocean zooplankton. First, if selection works efficiently in large populations, we would expect the assumption of neutrality to be violated often in studies of marine zooplankton. Indeed, we do commonly see significant deviations from neutrality across a broad array of planktonic taxa, with 16 of 21 studies reporting nonneutral evolution based on Tajima's *D*, Fu and Li's, or Rozas's *R*^2^ tests (001). However, it is well known that these commonly applied neutrality tests (e.g., Tajima's *D* test, Tajima 149) are sensitive to fluctuations in population size (e.g., Simonsen et al. 143; Nielsen 107), and significant results have often been interpreted in this context. The causative factors that underlie departures from neutrality therefore remain largely unknown, but could indicate that selection commonly influences the extent and distribution of genetic variation in marine zooplankton populations (see also Wares 161). Broader investigation of these patterns is warranted, and we call for consistent inclusion of rigorous tests of neutrality (see e.g., Nielsen 107 for a review) as a standard component of data analyses, such that it will be possible to assess how commonly the neutrality assumption is violated across taxa and loci. The McDonald–Kreitman (MK) test (Mcdonald and Kreitman 99) is particularly informative in cases where multiple protein-coding sequences of related species are available, because this test is robust to demographic assumptions (Nielsen 106). The MK test is based on the prediction of neutral theory that the ratio of replacement (nonsynonymous) to silent (synonymous) *fixed differences* observed between species should equal the ratio of replacement to silent *polymorphisms* within species. A significant excess of replacement fixed differences relative to silent changes is interpreted as evidence for adaptive evolution. To our knowledge, only one study of open ocean zooplankton has applied this test (Peijnenburg et al. 122), and it reported significant evidence for selection acting on the mitochondrial cytochrome oxidase II gene in two chaetognath species.

A second line of evidence suggesting that selection may be an important driver of evolution in species with large populations is the finding of large discrepancies between census and effective population sizes, which are commonplace for marine species (Grant and Bowen 69; Avise 6; Hauser and Carvalho 73; Portnoy et al. 129). Such discrepancies would be expected under widespread deviations from the neutral model. Studies of marine zooplankton that have contrasted census and effective population sizes, as estimated from abundance and genetic data, respectively, have reported dramatic differences ranging from 10^8^ to 10^10^ fold in chaetognaths (Peijnenburg et al. 122), copepods (Bucklin and Wiebe 23), and krill (Zane et al. 164; but see Zane et al. 165, reported a threefold difference). All of these studies used mitochondrial DNA to estimate effective population sizes, which, if under selection, would be expected to result in lower estimates of *N*_e_ relative to ‘true’ *N*_e_, as estimated from neutral markers. Recent work has also shown that a key prediction of the neutral theory, namely that species with large populations should have more genetic diversity than species with small populations, does not hold for mitochondrial DNA (Bazin et al. 9), and this observation was explained by the influence of pervasive selection on the mitochondrial genome (see also Meiklejohn et al. 101; Galtier et al. 58; Wares 161). Similarly for the nuclear genome, levels of genetic diversity across the tree of life do not scale with species abundances, which is inconsistent with a neutral model of evolution (Lynch 92; Hahn 71).

Third, and finally, as the strength and type of selection will differ between unlinked genetic markers, we would expect to see large differences in the degree of population structure detected by different loci if selection is an important driver of genetic differentiation (see also Fig. 2 and Piganeau et al. 128). Very few studies of zooplankton population structure have incorporated multiple unlinked genetic markers (001), making it difficult to evaluate how common such discrepancies are. One study that contrasted mitochondrial and nuclear variation in a planktonic chaetognath reported large differences in the degree of structure detected by the two marker types (Peijnenburg et al. 123). The historical heavy reliance on mtDNA markers in plankton population genetic studies has limited our ability to detect selection, and further tests of the ideas outlined above will become possible as the field moves toward genome-wide data and direct comparisons across loci and taxa. We do not yet have a representative view of the amounts of genomic diversity present in plankton populations and it remains to be tested whether selection models (and what types of model) or classical models of genetic drift are most appropriate to describe molecular evolution in zooplankton.

## Genetic Isolation in Open Ocean Habitats

One important supporting argument for the idea that zooplankton are slowly evolving is that high dispersal rates limit their capacity to evolve adaptively in response to spatially varying selection (e.g., Grosberg and Cunningham 70). But what is known regarding dispersal and connectivity among populations of open ocean zooplankton?

Early genetic research on the population structures of planktonic species often did support the view of nearly unlimited dispersal. Initially, workers focused on ecologically important, keystone species, and usually found genetic homogeneity among samples from distant areas of the distributional range. For example, *Euphausia superba*, the keystone euphausiid of the Antarctic pelagic ecosystem, has been a target species for population genetic studies dating back to the mid 1980s (e.g., 001; MacDonald et al. 94; Fevolden and Scheppenheim 56; Bortolotto et al. 17). Although a number of studies reported weak but significant population structure (Fevolden and Ayala 55; Zane et al. 164; Batta-Lona et al. 8), these patterns disappeared in more rigorous work with higher sample size and higher resolution genetic markers (Fevolden and Scheppenheim 56; Bortolotto et al. 17). Based on current evidence, it appears that this species is panmictic throughout its range. Note, however, that with even more powerful data (e.g., thousands of Single Nucleotide Polymorphism (SNP) loci accessed using Next Generation Sequencing technologies) it may well be that the null hypothesis of genetic homogeneity is ultimately rejected. Studies on *Calanus finmarchicus*, the dominant planktonic copepod in the boreal North Atlantic, followed a similar trajectory, with a number of reports of weak but significant differentiation between gyre systems and at basin spatial scales (001; Bucklin and Kocher 20; Bucklin et al. 28; Unal and Bucklin 154). However, using a powerful combination of mitochondrial and nuclear microsatellite loci, Provan et al. (131) demonstrated that the species lacks population genetic structure across the North Atlantic (Provan et al. 131). A number of studies on other holoplanktonic organisms also have hinted at high dispersal rates, including a notable series of studies on planktonic foraminifers that found identical genotypes across different ocean basins worldwide (de Vargas et al. 156, 157, 158, 159; note that isolation is also seen in this group, e.g., Aurahs et al. 5; Ujiie et al. 153; Seears et al. 141). In combination, this body of work has demonstrated that there probably *are* some holoplanktonic species that are panmictic throughout their distributional range. However, we argue here that the population structure of these hyper-abundant species may not be typical of the broader planktonic fauna, and an early focus on these species has inappropriately instilled the sense that holoplankton have universally high dispersal capacity.

The emerging patterns from genetic results on a broader array of species suggest a more complex and nuanced picture of dispersal and connectivity among populations of open ocean zooplankton. First, there is strong evidence for spatial genetic structure and limited gene flow among populations in a number of plankton species, with distributions ranging from coastal seas to open ocean habitats. For example, planktonic chaetognaths and copepods have been shown to have restricted dispersal in the coastal ocean (Peijnenburg et al. 121, 123; Papadopoulos et al. 118; Unal et al. 155; Chen and Hare 35; Yebra et al. 163), with rare multilocus studies providing the most compelling evidence for limited gene flow among European basins (Peijnenburg et al. 123; Reusch et al. 133). Truly oceanic species also have been found to harbor a number of genetically very distinct populations (Goetze 64, 67; Papetti et al. 119; Nelson et al. 105), with sample sizes sufficiently high to characterize the distribution of genetic variation at mesoscale (10s to 100s of km) to ocean basin spatial scales (>1000 km). If spatial genetic structure is interpreted in light of gene flow, these studies imply a much more limited dispersal range than expected for holoplanktonic organisms in open ocean habitats (e.g., Goetze 67). Nonmetazoan holoplankton also exhibit strong spatial genetic structure in a number of cases, implying that dispersal may be more limited than initially expected across a broad diversity of planktonic life (e.g., Darling et al. 45; McCauley et al. 97; Rynearson et al. 137; Casteleyn et al. 33; Ujiie et al. 153; Whittaker et al. 162).

A second emerging insight is that among zooplankton species with circumglobal or cosmopolitan biogeographic ranges, spatial genetic structure often occurs at the scale of pelagic biomes. For example, studies on the copepods *Eucalanus hyalinus*,* E. spinifer*,* Pleuromamma xiphias*, and *Haloptilus longicornis* all report genetic homogeneity among sample sites within subtropical gyres, with strong and significant population genetic breaks occurring between subtropical gyres and ocean basins (Goetze 64, 67; Norton and Goetze in press). These results imply limited dispersal across equatorial waters in all of these species, irrespective of whether or not the species is antitropical in distribution (*Eucalanus* species are antitropical, *Pleuromamma* and *Haloptilus* spp. are not). In these cases (and others), barriers to dispersal in the open sea occur at oceanographic regions with sharp transitions in both biotic and abiotic properties (Thornhill et al. 151; Goetze 67; Norton and Goetze in press), and are likely bio-physical in nature.

Third and finally, increasingly it appears that population genetic structure is species-specific in marine zooplankton, with closely related species often showing very different patterns of differentiation across the same pelagic habitats (Goetze 64; Blanco-Bercial et al. 16; Chen and Hare 35). These results suggest that there are differences between species in realized dispersal, which may be linked to species-specific ecological or habitat requirements. For example, Chen and Hare (35) documented salinity differences in the preferred habitat of three highly divergent lineages within *Acartia tonsa* that likely played a role in the development of phylogeographic structure within each of these cryptic species. The genetic lineage found in estuarine habitats showed high phylogeographic divergence among coastal embayments (US Atlantic coast), while the most oceanic lineage entirely lacked population genetic structure across the same sampling sites. Similar ecophysiological mechanisms likely control dispersal among populations of open ocean zooplankton, though there are no studies that have examined this for truly oceanic taxa. What constitutes optimal habitat will vary across species, with the nature of dispersal barriers highly dependent on the ecological niche of the organism. Therefore, it is more appropriate to consider population genetic structure a species-specific trait that varies across holoplanktonic taxa, as does any other ecological or life history trait, than to view the zooplankton as monotypically ‘high dispersal’ species.

This emerging view of the complexity of dispersal in marine zooplankton extends a number of observations that have been made in other marine species to the pelagic habitat. Panmixia was once expected for all marine species with large population size and a planktonic phase of the life history (e.g., Palumbi 116, 117). This idea now has been discarded as overly simplistic for both marine fish and benthic invertebrates (with meroplanktonic larvae), with extensive empirical studies having shown ‘closed’ marine populations and adaptive responses to human-induced and natural environmental change (e.g., Conover et al. 38; Hauser and Carvalho 73; Cowen and Sponaugle 39; Hellberg 76; Marshall et al. 96; Sanford and Kelly 138). There is now considerable evidence that many open ocean zooplankton species also demonstrate population genetic differentiation across distinct pelagic habitats, an observation that has important implications regarding their capacity to respond to local selective forces.

## New Views on Marine Zooplankton Evolution

Given the findings and arguments outlined above (on selection and genetic isolation), we hypothesize that open ocean zooplankton have large adaptive potential and may be capable of strong evolutionary responses (genetic adaptation) to changing ocean conditions. These responses could be rapid, occurring on ecological time scales (Hairston et al. 72; Schoener 140), and are thus important to consider in discussions about responses of the ocean's biota to contemporary climate change (Davis et al. 48; Parmesan 120). Significant findings of spatial population structuring in zooplankton (001) imply that selection could drive fitness toward a phenotypic optimum for ocean conditions experienced within a particular oceanographic habitat (e.g., a subtropical gyre), rather than across the range of environmental conditions that occur throughout the distribution of any particular species (often circumglobal in subtropical and tropical waters). This is important, as evolution can then trend toward specialization on specific ocean habitats rather than toward phenotypic plasticity or ecological generalism. In addition, marine zooplankton have short generation times, which can play a critical role in the rate of evolutionary change (e.g., Thomas et al. 150), and have been shown capable of very rapid evolution in peripheral marine environments (Dawson and Hamner 49) and during habitat invasions (Lee et al. 89, and references therein). These observations suggest that zooplankton can show rapid responses to selection under appropriate conditions.

One implication of our hypothesis is that marine zooplankton may have a more limited capacity to track suitable habitat under changing ocean conditions than is currently assumed (e.g., Provan et al. 131; Ji et al. 82; Reygondeau and Beaugrand 134). Results from the continuous plankton recorder (CPR) survey have convincingly demonstrated that range shifts are occurring at the northern boundary of the distribution of many temperate and boreal North Atlantic species in response to warming ocean conditions (Beaugrand et al. 12, 14; Beaugrand 10; Beaugrand and Reid 11). However, many of the species within the CPR record have distributions that extend across a range of pelagic biomes (e.g., subtropical gyres, boundary current, and equatorial upwelling ecosystems), and nothing is currently known regarding dispersal within and among different areas of their distribution. These species may in fact consist of a global collection of semi-isolated populations that respond individually to the changing distribution of their preferred habitat. Also, because the presence and strength of barriers to dispersal are expected to be species-specific for marine zooplankton (see above), the capacity of a species to track suitable habitat may vary across taxa and may lead to shifting community structure and trophic mismatches in pelagic foodwebs (e.g., as observed in the North Sea, Beaugrand et al. 13; Reygondeau and Beaugrand 134).

## Priorities for Future Research

Currently, very little is known about adaptation of zooplankton to local oceanographic conditions. Testing our hypothesis of high evolutionary potential in open ocean zooplankton will require research in at least two primary areas. First, the question of how pervasive natural selection is in driving population divergence in zooplankton needs to be addressed. Second, the spatial and temporal scales over which differentiation occurs in the open ocean need to be rigorously quantified. Next Generation sequencing (NGS) technologies have made possible the rapid generation of large-scale sequence data from nonmodel organisms at reasonable cost. We expect that these technologies (reviewed elsewhere in e.g., Davey et al. 47) and in particular Restriction-site associated DNA sequencing (RADSeq, Davey and Blaxter 46) will enable the field to move away from single marker studies (some fine examples of this approach include Emerson et al. 53; Hohenlohe et al. 79; Wagner et al. 160).

Indirect approaches can be used to address the question as to how pervasive selection is in driving the evolution of open ocean zooplankton (taxa that typically cannot be cultured in the lab). One cost-effective approach to examining adaptive variation in natural populations is to study functionally important traits related to, for instance, morphology or life history. Differentiation at these quantitative traits is typically expressed as *Q*_st_ or *P*_st_ (e.g., Merilä and Crnokrak 102; McKay and Latta 100; Leinonen et al. 90) and can be compared to patterns of differentiation at neutral genetic markers, which are typically expressed as *F*_st_. If *Q*_st_ > *F*_st_, diversifying selection is invoked, in which different phenotypes are favored in different populations. Whereas if *Q*_st_ < *F*_st_, this suggests stabilizing selection, and the same phenotype is favored across populations. Another indirect approach would be to use genome scans to contrast adaptive and neutral variation across the genome (e.g., reviewed in Davey et al. 47). By comparing divergent populations, it is possible to identify loci showing signatures of selection using, for instance, outlier analyses, cline analyses, or tests of selection based on ratios of neutral and adaptive changes (reviewed in e.g., Nielsen 107; Nielsen et al. 108; Rice et al. 135). A third indirect approach is the candidate gene approach, in which specific loci (genes) of known functional significance are sequenced. A nice example is provided by Larmuseau et al. (88) in which a sensory gene, rhodopsin, was sequenced for sand goby individuals from different populations in Europe. They found that sequences of this gene did not cluster according to geographical or historical proximity, but according to the general photic conditions of the habitat of the fish. Zooplankton populations sampled along naturally occurring gradients of temperature, salinity, or acidification, which change with latitude and/or depth, could be examined for sequence variation at neutral as well as targeted genetic loci. An example of a genome-wide approach can be found in Bradbury et al. (18), in which a survey of single nucleotide polymorphisms in Atlantic cod was used to pinpoint 40 loci for which allele frequencies showed parallel temperature-associated clines in the eastern and western north Atlantic. Note that the effect of selection is inferred indirectly using these methods and additional experiments (e.g., laboratory, mapping, and/or gene expression studies) would be necessary to link genetic loci with specific targets of selection (see Feder and Mitchell-Olds' 54 perspective on the emerging field of evolutionary and ecological functional genomics [EEFG] which seeks to understand the functional basis of evolutionary forces shaping ecologically important traits in natural biological communities). Once several comparative genome-wide datasets become available from natural populations of planktonic taxa, we can begin to draw more general conclusions about the levels of intraspecific and intragenomic genetic variability and the relative importance of selection.

In order to rigorously quantify the spatial and temporal scales over which differentiation occurs in the open ocean, we need to substantially increase the types and amounts of data that are collected. First, it is critically important to be able to compare genetic results across marker types in order to obtain rigorous estimates of dispersal through Bayesian, Maximum Likelihood, or coalescent analytical techniques (e.g., Pritchard et al. 130; Hey and Nielsen 78; Zhang 166; Beerli and Palczewski 15; Hey 77; Nielsen et al. 109). The historical dominance of single marker studies has limited our capacity to distinguish demographic forces (migration, changing population size) from selection. Second, limited sampling in terms of the number of individuals sampled per population (001), but also across space and time, has resulted in low scientific rigor in some studies. Revealing the amounts of genetic variation present within populations is particularly important, because this is a critical component to assessing evolutionary potential (e.g., Chevin et al. 36). In addition, temporal variability needs to be more comprehensively addressed in future work, as nearly nothing is known regarding the stability of zooplankton population structure through time (but see Peijnenburg et al. 123 and Bortolotto et al. 17 as rare examples of studies with time-stratified sampling). Third, and finally, resolving spatial patterns of gene flow for a greater diversity of species is also paramount to understanding the nature of dispersal barriers for marine zooplankton. Current evidence suggests that realized dispersal for holoplanktonic species may be linked to the ecological niche of the organism, and this idea needs to be tested with community-wide data on population genetic structures of a diverse panel of species with distinct pelagic niches. Ideally, such species should be collected on the same research cruises so that comparable spatial and temporal scales are sampled. Another approach would be to compare population genetic structures of related species with different life history or ecological characteristics as is common in the benthic invertebrates research community.

Finally, a wide range of questions on the fundamental basis of evolutionary change in marine zooplankton remain to be addressed. We hope that the ideas outlined here, in combination with the suite of high-throughput genomics techniques now available for development of genome-wide markers in nonmodel species will catalyze research in this field.

## References

[b1] Afanas'yev KI, Flint MV, Fetisov AN (1989). Population genetics of two mass species of Pacific ocean copepods. Oceanology.

[b2] Angel MV, Peterson NA (1992). Pelagic biodiversity. Diversity of oceanic life: an evaluative review.

[b3] Angel MV (1993). Biodiversity of the pelagic ocean. Conserv. Biol.

[b4] Appeltans W, Ahyong ST, Anderson G, Angel MV, Artois T, Bailly N (2012). The magnitude of global marine species diversity. Curr. Biol.

[b5] Aurahs R, Grimm GW, Hemleben V, Hemleben C, Kucera M (2009). Geographical distribution of cryptic genetic types in the planktonic foraminifer *Globigerinoides ruber*. Mol. Ecol.

[b6] Avise JC (2000). Phylogeography: the history and formation of species.

[b7] Barton N (2010). Understanding adaptation in large populations. PLoS Genet.

[b8] Batta-Lona PG, Bucklin A, Weibe PH, Patarnello T, Copley NJ (2011). Population genetic variation of the Southern Ocean krill, *Euphausia superba*, in the Western Antarctic Peninsula region based on mitochondrial single nucleotide polymorphisms (SNP). Deep Sea Res. Part II.

[b9] Bazin E, Glemin S, Galtier N (2006). Population size does not influence mitochondrial genetic diversity in animals. Science.

[b10] Beaugrand G (2003). Long-term changes in copepod abundance and diversity in the north-east Atlantic in relation to fluctuations in the hydroclimatic environment. Fish. Oceanogr.

[b11] Beaugrand G, Reid PC (2003). Long-term changes in phytoplankton, zooplankton and salmon related to climate. Glob. Change Biol.

[b12] Beaugrand G, Reid PC, Ibañez F, Lindley JA, Edwards M (2002). Reorganization of North Atlantic marine copepod biodiversity and climate. Science.

[b13] Beaugrand G, Brander KM, Lindley JA, Souissi S, Reid PC (2003). Plankton effect on cod recruitment in the North Sea. Nature.

[b14] Beaugrand G, Luczak C, Edwards M (2009). Rapid biogeographical plankton shifts in the North Atlantic Ocean. Glob. Change Biol.

[b15] Beerli P, Palczewski M (2010). Unified framework to evaluate panmixia and migration direction among multiple sampling locations. Genetics.

[b16] Blanco-Bercial L, Alvarez-Marquez F, Bucklin A (2011). Comparative phylogeography and connectivity of sibling species of the marine copepod *Clausocalanus* (Calanoida). J. Exp. Mar. Biol. Ecol.

[b17] Bortolotto E, Bucklin A, Mezzavilla M, Zane L, Patarnello T (2011). Gone with the currents: lack of genetic differentiation at the circum-continental scale in Antarctic krill *Euphausia superba*. BMC Genet.

[b18] Bradbury IR, Hubert S, Higgins B, Borza T, Bowman S, Paterson IG (2010). Parallel adaptive evolution of Atlantic cod on both sides of the Atlantic Ocean in response to temperature. Proc. Biol. Sci.

[b19] Bucklin A (1991). Population genetic responses of the planktonic copepod *Metridia pacifica* to a coastal eddy in the California Current. J. Geophys. Res.

[b20] Bucklin A, Kocher TD (1996). Source regions for recruitment of *Calanus finmarchicus* to Georges Bank: evidence from molecular population genetic analysis of mtDNA. Deep Sea Res. Part II.

[b21] Bucklin A, Marcus NH (1985). Genetic differentiation of populations of the planktonic copepod *Labidocera aetiva*. Mar. Biol.

[b22] Bucklin A, Wiebe PH (1986). Genetic heterogeneity in euphausiid populations–*Euphausia krohnii* and *Nematoscelis megalops* in North-Atlantic slope water. Limnol. Oceanogr.

[b23] Bucklin A, Wiebe PH (1998). Low mitochondrial diversity and small effective population sizes of the copepods *Calanus finmarchicus* and *Nannocalanus minor:* possible impact of climatic variation during recent glaciation. J. Hered.

[b24] Bucklin A, Rienecker MM, Mooers CNK (1989). Genetic tracers of zooplankton transport in coastal filaments off northern California. J. Geophys. Res.

[b25] Bucklin A, Sundt RC, Dahle G (1996a). The population genetics of *Calanus finmarchicus* in the North Atlantic. Ophelia.

[b26] Bucklin A, LaJeunesse TC, Curry E, Wallinga J, Garrison K (1996b). Molecular diversity of the copepod, *Nannocalanus minor*: genetic evidence of species and population structure in the North Atlantic Ocean. J. Mar. Res.

[b27] Bucklin A, Smolenack SB, Bentley AM, Wiebe PH (1997). Gene flow patterns of the euphausiid, *Meganyctiphanes norvegica*, in the NW Atlantic based on mtDNA sequences for cytochrome b and cytochrome oxidase I. J. Plankton Res.

[b28] Bucklin A, Astthorsson OS, Gislason A, Allen LD, Smolenack SB, Wiebe PH (2000a). Population genetic variation in *Calanus finmarchicus* in Icelandic waters: preliminary evidence of genetic differences between Atlantic and Arctic populations. ICES J. Mar. Sci.

[b29] Bucklin A, Kaartvedt S, Guarnieri M, Goswami U (2000b). Population genetics of drifting (*Calanus* spp.) and resident (*Acartia clausi*) plankton in Norwegian fjords. J. Plankton Res.

[b30] Bucklin A, Wiebe PH, Smolenack SB, Copley NJ, Clarke ME (2002). Integrated biochemical, molecular genetic, and bioacoustical analysis of mesoscale variability of the euphausiid *Nematoscelis difficilis* in the California Current. Deep Sea Res. Part I.

[b31] Bucklin A, Steinke D, Blanco-Bercial L (2011). DNA barcoding of marine metazoa. Annu. Rev. Mar. Sci.

[b32] Burrows MT, Schoeman DS, Buckley LB, Moore P, Poloczanska ES, Brander KM (2011). The pace of shifting climate in marine and terrestrial ecosystems. Science.

[b33] Casteleyn G, Leliaert F, Backeljau T, Debeer A-E, Kotaki Y, Rhodes L (2010). Limits to gene flow in a cosmopolitan marine planktonic diatom. Proc. Natl. Acad. Sci.

[b34] Charlesworth B (2009). Effective population size and patterns of molecular evolution and variation. Nat. Rev. Genet.

[b35] Chen G, Hare MP (2011). Cryptic diversity and comparative phylogeography of the estuarine copepod *Acartia tonsa* on the US Atlantic coast. Mol. Ecol.

[b36] Chevin LM, Lande R, Mace GM (2010). Adaptation, plasticity, and extinction in a changing environment: towards a predictive theory. PLoS Biol.

[b37] Colin SP, Dam HG (2007). Comparison of the functional and numerical responses of resistant versus non-resistant populations of the copepod *Acartia hudsonica* fed the toxic dinofiagellate *Alexandrium tamarense*. Harmful Algae.

[b38] Conover DO, Clarke LM, Munch SB, Wagner GN (2006). Spatial and temporal scales of adaptive divergence in marine fishes and the implications for conservation. J. Fish Biol.

[b39] Cowen RK, Sponaugle S (2009). Larval dispersal and marine population connectivity. Annu. Rev. Mar. Sci.

[b40] Coyne JA, Orr HA (2004). Speciation.

[b41] Critescu ME, Constantin A, Bock DG, Cáceres CE, Crease TJ (2012). Speciation with gene flow and the genetics of habitat transitions. Mol. Ecol.

[b42] Crow JF, Kimura M (1970). An introduction to population genetics theory.

[b43] Dam HG (2013). Evolutionary adaptation of marine zooplankton to global change. Annu. Rev. Mar. Sci.

[b44] Darling KF, Wade CA (2008). The genetic diversity of planktic foraminifera and the global distribution of ribosomal RNA genotypes. Mar. Micropaleontol.

[b45] Darling KF, Kucera M, Wade CM (2007). Global molecular phylogeography reveals persistent Arctic circumpolar isolation in a marine planktonic protist. Proc. Natl. Acad. Sci.

[b46] Davey JW, Blaxter ML (2011). RADSeq: next-generation population genetics. Brief. Funct. Genomics.

[b47] Davey JW, Hohenlohe PA, Etter PD, Boone JQ, Catchen JM, Blaxter ML (2011). Genome-wide genetic marker discovery and genotyping using next-generation sequencing. Nat. Rev. Genet.

[b48] Davis MB, Shaw RG, Etterson JR (2005). Evolutionary responses to changing climate. Ecology.

[b49] Dawson MN, Hamner WM (2005). Rapid evolutionary radiation of marine zooplankton in peripheral environments. Proc. Natl. Acad. Sci. USA.

[b50] Dawson MN, Jacobs DK (2001). Molecular evidence for cryptic species of *Aurelia aurita* (Cnidaria, Scyphozoa). Biol. Bull.

[b51] Eberl R, Cohen S, Cipriano F, Carpenter EJ (2007). Genetic diversity of the pelagic harpacticoid copepod *Macrosetella gracilis* on colonies of the cyanobacterium *Trichodesmium* spp. Aquat. Biol.

[b52] Ekman S (1953). Zoogeography of the sea.

[b53] Emerson KJ, Merz CR, Catchen JM, Hohenlohe PA, Cresko WA, Bradshaw WE (2010). Resolving postglacial phylogeography using high-throughput sequencing. Proc. Natl Acad. Sci. USA.

[b54] Feder ME, Mitchell-Olds TM (2003). Evolutionary and ecological functional genomics. Nat. Rev. Genet.

[b55] Fevolden SE, Ayala FJ (1981). Enzyme polymorphism in Antarctic krill (Euphausiacea); genetic variation between populations and species. Sarsia.

[b56] Fevolden SE, Scheppenheim R (1989). Genetic homogeneity of krill (*Euphausia superba* Dana) in the Southern Ocean. Polar Biol.

[b57] Gaggiotti OE, Bekkevold D, Jorgensen HBH, Foll M, Carvalho GR, Andre C (2009). Disentangling the effects of evolutionary, demographic, and environmental factors influencing genetic structure of natural populations: Atlantic herring as a case study. Evolution.

[b58] Galtier N, Nabholz B, Glemin S, Hurst GDD (2009). Mitochondrial DNA as a marker of molecular diversity: a reappraisal. Mol. Ecol.

[b59] Gerrish PJ, Lenski RE (1998). The fate of competing beneficial mutations in an asexual population. Genetica.

[b60] Gillespie JH (1991). The causes of molecular evolution.

[b61] Gillespie JH (1999). The role of population size in molecular evolution. Theor. Popul. Biol.

[b62] Gillespie JH (2000). Genetic drift in an infinite population: the pseudohitchhiking model. Genetics.

[b63] Gillespie JH (2001). Is the population size of a species relevant to its evolution?. Evolution.

[b64] Goetze E (2005). Global population genetic structure and biogeography of the oceanic copepods *Eucalanus hyalinus* and *E. spinifer*. Evolution.

[b65] Goetze E (2010a). Species discovery through large-scale molecular screening in the planktonic copepod family Eucalanidae. Mol. Ecol.

[b66] Goetze E (2010b). Integrated molecular and morphological biogeography of the calanoid copepod family Eucalanidae. Deep Sea Res. Part II.

[b67] Goetze E (2011). Population differentiation in the open sea: insights from the pelagic copepod *Pleuromamma xiphias*. Integr. Comp. Biol.

[b68] Goodall-Copestake WP, Perez-Espona S, Clark MS, Murphy EJ, Seear PJ, Tarling GA (2010). Swarms of diversity at the gene cox1 in Antarctic krill. Heredity.

[b69] Grant WS, Bowen BW (1998). Shallow population histories in deep evolutionary lineages of marine fishes: insights from sardines and anchovies and lessons for conservation. J. Hered.

[b70] Grosberg RK, Cunningham CW, Bertness MD, Gaines SD, Hay ME (2001). Genetic structure in the sea: from populations to communities. Marine community ecology.

[b71] Hahn MW (2008). Toward a selection theory of molecular evolution. Evolution.

[b72] Hairston NG, Ellner SP, Geber MA, Yoshida T, Fox JA (2005). Rapid evolution and the convergence of ecological and evolutionary time. Ecol. Lett.

[b73] Hauser L, Carvalho GR (2008). Paradigm shifts in marine fisheries genetics: ugly hypotheses slain by beautiful facts. Fish Fish.

[b74] Hays GC, Richardson AJ, Robinson C (2005). Climate change and marine plankton. Trends Ecol. Evol.

[b75] Helaouët P, Beaugrand G (2009). Physiology, ecological niches and species distributions. Ecosystems.

[b76] Hellberg ME (2009). Gene flow and isolation among populations of marine animals. Annu. Rev. Ecol. Evol. Syst.

[b77] Hey J (2010). Isolation with migration models for more than two populations. Mol. Biol. Evol.

[b78] Hey J, Nielsen R (2004). Multilocus methods for estimating population sizes, migration rates and divergence time, with applications to the divergence of *Drosophila pseudoobscura* and *D. persimilis*. Genetics.

[b79] Hohenlohe PA, Bassham S, Etter PD, Stiffler N, Johnson EA, Cresko WA (2010). Population genomics of parallel adaptation in threespine stickleback using sequenced RAD tags. PLoS Genet.

[b80] Jarman SN, Elliott NG, Nicol S, McMinn A (2002). Genetic differentiation in the Antarctic coastal krill *Euphausia crystallorophias*. Heredity.

[b81] Jennings RM, Bucklin A, Ossenbrugger H, Hopcroft RR (2010). Species diversity of planktonic gastropods (Pteropoda and Heteropoda) from six ocean regions based on DNA barcode analysis. Deep Sea Res. Part II.

[b82] Ji R, Edwards M, Mackas DL, Runge JA, Thomas AC (2010). Marine plankton phenology and life history in a changing climate: current research and future directions. J. Plankton Res.

[b83] Kann LM, Wishner K (1996). Genetic population structure of the copepod *Calanus finmarchicus* in the Gulf of Maine: allozyme and amplified mitochondrial DNA variation. Mar. Biol.

[b84] Karasov T, Messer PW, Petrov DA (2010). Evidence that adaptation in Drosophila is not limited by mutation at single sites. PLoS Genet.

[b85] Kimura M (1968). Evolutionary rate at the molecular level. Nature.

[b86] Kimura M (1983). The neutral theory of molecular evolution.

[b87] Kuhl S, Schneppenheim R (1986). Electrophoretic investigation of genetic-variation in 2 krill species *Euphausia superba* and *Euphausia crystallorophias* (Euphausiidae). Polar Biol.

[b88] Larmuseau MHD, Raeymaekers JAM, Ruddick KG, Volckaert JKJ, van Houdt FAM (2009). To see in different seas: spatial variation in the rhodopsin gene of the sand goby (*Pomatoschistus minutus*. Mol. Ecol.

[b89] Lee CE, Kiergaard M, Gelembiuk GW, Eads BD, Posavi M (2011). Pumping ions: rapid parallel evolution of ionic regulation following habitat invasions. Evolution.

[b90] Leinonen T, O'Hara RB, Cano JM, Merilä J (2008). Comparative studies of quantitative trait and neutral marker divergence: a meta-analysis. J. Evol. Biol.

[b91] Lohbeck KT, Riebesell U, Reusch TBH (2012). Adaptive evolution of a key phytoplankton species to ocean acidification. Nat. Geosci.

[b92] Lynch M (2006). The origins of eukaryotic gene structure. Mol. Biol. Evol.

[b93] Lynch M, Gabriel W, Wood AM (1991). Adaptive and demographic responses of plankton populations to environmental change. Limnol. Oceanogr.

[b94] MacDonald CM, Williams RL, Adams M (1986). Genetic variation and population structure of krill (*Euphausia superba* Dana) from the Prydz Bay region of Antarctic waters. Polar Biol.

[b95] Machida RJ, Nishida S (2010). Amplified fragment length polymorphism analysis of the mesopelagic copepod *Disseta palumbii* in the equatorial western Pacific and adjacent waters: role of marginal seas in the genetic isolation of mesopelagic animals. Deep Sea Res. Part II.

[b96] Marshall DJ, Monro K, Bode M, Keough MJ, Swearer S (2010). Phenotype-environment mismatches reduce connectivity in the sea. Ecol. Lett.

[b97] McCauley LAR, Erdner DL, Nagai S, Richlen ML, Anderson DM (2009). Biogeographic analysis of the globally distributed harmful algal bloom species *Alexandrium Minutum* (Dinophyceae) based on rRNA gene sequences and microsatellite markers. J. Phycol.

[b98] McCusker MR, Bentzen P (2010). Positive relationships between genetic diversity and abundance in fishes. Mol. Ecol.

[b99] Mcdonald JH, Kreitman M (1991). Adaptive protein evolution at the Adh locus in Drosophila. Nature.

[b100] McKay JK, Latta RG (2002). Adaptive population divergence: markers, QTL and traits. Trends Ecol. Evol.

[b101] Meiklejohn CD, Montooth KL, Rand DM (2007). Positive and negative selection on the mitochondrial genome. Trends Genet.

[b102] Merilä J, Crnokrak P (2001). Comparison of genetic differentiation at marker loci and quantitative traits. J. Evol. Biol.

[b103] Miyamoto H, Machida RJ, Nishida S (2010). Genetic diversity and cryptic speciation of the deep sea chaetognath *Caecosagitta macrocephala* (Fowler, 1904). Deep Sea Res. Part II.

[b104] Morard R, Quillevere F, Escarguel G, Ujiie Y, Norris T, de Garidel-Thoron RD (2009). Morphological recognition of cryptic species in the planktonic foraminifer *Orbulina universa*. Mar. Micropaleontol.

[b105] Nelson RJ, Carmack EC, McLaughlin FA, Cooper GA (2009). Penetration of Pacific zooplankton into the western Arctic Ocean tracked with molecular population genetics. Mar. Ecol. Prog. Ser.

[b106] Nielsen R (2001). Statistical tests of selective neutrality in the age of genomics. Heredity.

[b107] Nielsen R (2005). Molecular signatures of natural selection. Annu. Rev. Genet.

[b108] Nielsen EE, Hemmer-Hansen J, Larsen PF, Bekkevold D (2009). Population genomics of marine fishes: identifying adaptive variation in space and time. Mol. Ecol.

[b109] Nielsen R, Korneliussen T, Albrechtsen A, Li Y, Wang J (2012). SNP calling, genotype calling, and sample allele frequency estimation from New-Generation Sequencing data. PloS ONE.

[b110] Norris RD (2000). Pelagic species diversity, biogeography, and evolution. Paleobiology.

[b111] Norris RD, de Vargas C (2000). Evolution all at sea. Nature.

[b112] Norton EL, Goetze E Equatorial dispersal barriers and limited population connectivity among oceans in a planktonic copepod. Limnol. Oceanogr.

[b113] Nuwer ML, Frost B, Armbrust EV (2008). Population structure of the planktonic copepods *Calanus pacificus* in the North Pacific Ocean. Mar. Biol.

[b114] Orsini L, Spanier KI, de Meester L (2012). Genomic signature of natural and anthropogenic stress in wild populations of the waterflea *Daphnia magna*: validation in space, time and experimental evolution. Mol. Ecol.

[b115] Ortman BD, Bucklin A, Pages F, Youngbluth M (2010). DNA barcoding the Medusozoa using mtCOI. Deep Sea Res. Part II.

[b116] Palumbi SR (1992). Marine speciation on a small planet. Trends Ecol. Evol.

[b117] Palumbi SR (1994). Genetic divergence, reproductive isolation, and marine speciation. Annu. Rev. Ecol. Syst.

[b118] Papadopoulos LN, Peijnenburg KTCA, Luttikhuizen PC (2005). Phylogeography of the calanoid copepods *Calanus helgolandicus* and *C. euxinus* suggests Pleistocene divergences between Atlantic, Mediterranean, and Black Sea populations. Mar. Biol.

[b119] Papetti C, Zane L, Bortolotto E, Bucklin A, Patarnello T (2005). Genetic differentiation and local temporal stability of population structure in the euphausiid *Meganyctiphanes norvegica*. Mar. Ecol. Prog. Ser.

[b120] Parmesan C (2006). Ecological and evolutionary responses to recent climate change. Annu. Rev. Ecol. Evol. Syst.

[b121] Peijnenburg KTCA, Breeuwer JAJ, Pierrot-Bults AC, Menken SBJ (2004). Phylogeograpy of the planktonic chaetognath *Sagitta setosa* reveals isolation in European seas. Evolution.

[b122] Peijnenburg KTCA, Fauvelot EK, van Haastrecht C (2005). Present-day genetic composition suggests contrasting demographic histories of two dominant chaetognaths of the North-East Atlantic, *Sagitta elegans* and *S. setosa*. Mar. Biol.

[b123] Peijnenburg KTCA, Fauvelot C, Breeuwer JAJ, Menken SBJ (2006). Spatial and temporal genetic structure of the planktonic *Sagitta setosa* (Chaetognatha) in European seas as revealed by mitochondrial and nuclear DNA markers. Mol. Ecol.

[b124] Pelejero C, Calvo E, Hoegh-Guldberg O (2010). Paleo-perspectives on ocean acidification. Trends Ecol. Evol.

[b125] Pennings PS, Hermisson J (2006a). Soft sweeps II-molecular population genetics of adaptation from recurrent mutation or migration. Mol. Biol. Evol.

[b126] Pennings PS, Hermisson J (2006b). Soft sweeps III: the signature of positive selection from recurrent mutation. PLoS Genet.

[b127] Pierrot-Bults AC, Pierrot-Bults S, van der Spoel L, van der Spoel SAC (1979). Speciation in macrozooplankton. Zoogeography and diversity of plankton.

[b128] Piganeau G, Eyre-Walker A, Grimsley N, Moreau H (2011). How and why DNA barcodes underestimate the diversity of microbial eukaryotes. PLoS ONE.

[b129] Portnoy DS, McDowell JR, McCandless CT, Musick JA, Graves JE (2009). Effective size closely approximates the census size in the heavily exploited western Atlantic population of the sandbar shark, *Carcharhinus plumbeus*. Conserv. Genet.

[b130] Pritchard JK, Stephens M, Donnelly P (2000). Inference of population structure using multilocus genotype data. Genetics.

[b131] Provan J, Beatty GE, Keating SL, Maggs CA, Savidge G (2009). High dispersal potential has maintained long-term population stability in the North Atlantic copepod *Calanus finmarchicus*. Proc. Biol. Sci.

[b132] Ralph P, Coop G (2010). Parallel adaptation: one or many waves of advance of an advantageous allele?. Genetics.

[b133] Reusch TBH, Bolte S, Sparwel M, Moss AG, Javidpour J (2010). Microsatellites reveal origin and genetic diversity of Eurasian invasions by one of the world's most notorious marine invader, *Mnemiopsis leidyi* (Ctenophora). Mol. Ecol.

[b134] Reygondeau G, Beaugrand G (2010). Future climate-driven shifts in distribution of *Calanus finmarchicus*. Glob. Change Biol.

[b135] Rice AM, Rudh A, Ellegren H, Qvarnstrom A (2011). A guide to the genomics of ecological speciation in natural animal populations. Ecol. Lett.

[b136] Richardson AJ (2008). In hot water: zooplankton and climate change. ICES J. Mar. Sci.

[b137] Rynearson TA, Lin EO, Armbrust EV (2009). Metapopulation structure in the planktonic diatom *Ditylum brightwellii* (Bacillariophyceae). Protist.

[b138] Sanford E, Kelly MW (2011). Local adaptation in marine invertebrates. Annu. Rev. Mar. Sci.

[b139] Schneppenheim R, Macdonald CM (1984). Genetic variation and population structure of krill (*Euphausia superba*) in the Atlantic sector of Antarctic waters and off the Antarctic Peninsula. Polar Biol.

[b140] Schoener TW (2011). The newest synthesis: understanding the interplay of evolutionary and ecological dynamics. Science.

[b141] Seears HA, Darling KF, Wade CM (2012). Ecological partitioning and diversity in tropical planktonic foraminifera. BMC Evol. Biol.

[b142] Sexton PF, Norris RD (2008). Dispersal and biogeography of marine plankton: long-distance dispersal of the foraminifer *Truncorotalia truncatulinoides*. Geology.

[b143] Simonsen KL, Churchill GA, Aquadro CF (1995). Properties of statistical tests of neutrality for DNA polymorphism data. Genetics.

[b144] van der Spoel S (1994). The basis for boundaries in pelagic biogeography. Prog. Oceanogr.

[b145] van der Spoel S, Pierrot-Bults AC (1979). Zoogeography and diversity of plankton.

[b146] Stegert C, Ji R, Davis CS (2010). Influence of projected ocean warming on population growth potential in two North Atlantic copepod species. Prog. Oceanogr.

[b147] Stopar K, Ramsak A, Tronteij P, Malej A (2010). Lack of genetic structure in the jellyfish *Pelagia noctiluca* (Cnidaria: Scyphozoa: Semaeostomeae) across European seas. Mol. Phylogenet. Evol.

[b148] Sundt RC, Fevolden SE (1996). Homogeneous genetic structure of *Meganyctiphanes norvegica* (Euphausiacea) in the north-east Atlantic Ocean, as interpreted from allozymic variation. Sarsia.

[b149] Tajima F (1989). The effect of change in population size on DNA polymorphism. Genetics.

[b150] Thomas JA, Welch JJ, Lanfear R, Bromham L (2010). A generation time effect on the rate of molecular evolution in invertebrates. Mol. Biol. Evol.

[b151] Thornhill DJ, Mahon AR, Norenburg JL, Halanych KM (2008). Open-ocean barriers to dispersal: a test case with the Antarctic Polar Front and the ribbon worm *Parborlasia corrugatus* (Nemteria: Lineidae). Mol. Ecol.

[b152] Thuesen EV, Numachi K, Nemoto T (1993). Genetic variation in the planktonic chaetognaths *Parasagitta elegans* and *Eukrohnia hamata*. Mar. Ecol. Prog. Ser.

[b153] Ujiie Y, Asami T, Liu T, de Garidel-Thoron H, Ishitani Y, de Vargas C (2012). Longitudinal differentiation among pelagic populations in a planktic foraminifer. Ecol. Evol.

[b154] Unal E, Bucklin A (2010). Basin-scale population genetic structure of the planktonic copepod *Calanus finmarchicus* in the North Atlantic Ocean. Prog. Oceanogr.

[b155] Unal E, Frost BW, Armbrust EV, Kideys AE (2006). Phylogeography of *Calanus helgolandicus* and the Black Sea copepod *Calanus euxinus*, with notes on *Pseudocalanus elongatus* (Copepoda: Calanoida). Deep Sea Res. Part II.

[b156] de Vargas C, Norris R, Zaninetti L, Gibb SW, Pawlowski J (1999). Molecular evidence of cryptic speciation in planktonic foraminifers and their relation to oceanic provinces. Proc. Natl. Acad. Sci. USA.

[b157] de Vargas C, Renaud S, Hilbrecht H, Pawlowski J (2001). Pleistocene adaptive radiation in *Globorotalia truncatulinoides*: genetic, morphologic, and environmental evidence. Paleobiology.

[b158] de Vargas C, Bonzon M, Rees NW, Pawlowski J, Zaninetti L (2002). A molecular approach to biodiversity and biogeography in the planktonic foraminifer *Globigerinella siphonifera* (d/Orbigny). Mar. Micropaleontol.

[b159] de Vargas C, Saez AG, Medlin LK, Thierstein HR, Theirstein HR, Young JR (2004). Super-species in the calcareous plankton. Coccolithophores: from molecular processes to global impact.

[b160] Wagner CE, Keller I, Wittwer S, Selz OM, Mwaiko S, Greuter L (2013). Genome-wide RAD sequence data provide unprecedented resolution of species boundaries and relationships in the Lake Victoria cichlid adaptive radiation. Mol. Ecol.

[b161] Wares JP (2010). Natural distributions of mitochondrial sequence diversity support new null hypotheses. Evolution.

[b162] Whittaker KA, Rignanese DR, Olson RJ, Rynearson TA (2012). Molecular subdivision of the marine diatom *Thalassiosira rotula* in relation to geographic distribution, genome size, and physiology. BMC Evol. Biol.

[b163] Yebra L, Bonnet D, Harris RP, Lindeque PK, Peijnenburg KTCA (2011). Barriers in the pelagic: population structuring of *Calanus helgolandicus* and *C. euxinus* in European waters. Mar. Ecol. Prog. Ser.

[b164] Zane L, Ostellari L, Maccatrozzo L, Bargelloni L, Battaglia B, Patarnello T (1998). Molecular evidence for genetic subdivision of Antarctic krill (*Euphausia superba* Dana) populations. Proc. Biol. Sci.

[b165] Zane L, Ostellari L, Maccatrozzo L, Bargelloni L, Cuzin-Roudy J, Buchholz F (2000). Genetic differentiation in a pelagic crustacean (*Meganyctiphanes norvegica*, Euphausiacea) from the North East Atlantic and the Meditteranean Sea. Mar. Biol.

[b166] Zhang Y (2008). Tree-guided Bayesian inference of population structures. Bioinformatics.

